# Restoration of Aqueous Humor Outflow Following Transplantation of iPSC-Derived Trabecular Meshwork Cells in a Transgenic Mouse Model of Glaucoma

**DOI:** 10.1167/iovs.16-20672

**Published:** 2017-04

**Authors:** Wei Zhu, Ankur Jain, Oliver W. Gramlich, Budd A. Tucker, Val C. Sheffield, Markus H. Kuehn

**Affiliations:** 1 Department of Ophthalmology and Visual Sciences, University of Iowa, Iowa City, Iowa, United States; 2 Center for the Prevention and Treatment of Visual Loss, Iowa City Veterans Affairs Medical Center, Iowa City, Iowa, United States; 3 Department of Pediatrics, University of Iowa, Iowa City, Iowa, United States; 4 Howard Hughes Medical Institute, University of Iowa College of Medicine, Iowa City, Iowa, United States

**Keywords:** iPSC, regenerative medicine, trabecular meshwork

## Abstract

**Purpose:**

Primary open-angle glaucoma (POAG) is particularly common in older individuals and associated with pathologic degeneration of the trabecular meshwork (TM). We have shown previously that transplantation of induced pluripotent stem cell (iPSC) derived TM cells restores aqueous humor dynamics in young transgenic mice expressing a pathogenic form of human myocilin (Tg-MYOC^Y437H^). This study was designed to determine if this approach is feasible in older mice with more pronounced TM dysfunction.

**Methods:**

Mouse iPSC were differentiated toward a TM cell phenotype (iPSC-TM) and injected into the anterior chamber of 6-month-old Tg-MYOC^Y437H^ or control mice. IOP and aqueous humor outflow facility were recorded for up to 3 months. Transmission electron microscopy, Western blot, and immunohistochemistry were performed to analyze TM morphology, quantify endoplasmic reticulum (ER) stress, and assess TM cellularity.

**Results:**

A 12 weeks after transplantation, IOP in iPSC-TM recipients was statistically lower and outflow facility was significantly improved compared to untreated controls. The number of endogenous TM cells increased significantly in iPSC-TM recipients along with the appearance of TM cells immmunopositive for a marker of cellular division. Morphologically, transplantation of iPSC-TM preserves ER structure 12 weeks after transplantation. However, myocilin and calnexin expression levels remain elevated in transplanted eyes of these 9-month-old Tg-MYOC^Y437H^ mice, indicating that ER stress persists within the TM.

**Conclusions:**

Transplantation of iPSC-TM can restore IOP and outflow facility in aged Tg-MYOC^Y437H^ mice. This type of stem cell–based therapy is a promising possibility for restoration of IOP control in some glaucoma patients.

Although it is not strictly correlated with the development of primary open-angle glaucoma (POAG), elevated IOP is an important risk factor for the disease.^1,2^ The IOP is the result of the balance between aqueous humor production by the ciliary body and the ability of the ocular outflow pathways to remove this fluid, particularly the trabecular meshwork (TM) and the endothelium of Schlemm's canal.^3,4^ Reduction of IOP through medical or surgical means remains the only approach proven to slow or halt the progression of glaucoma, but losses in aqueous humor outflow facility currently cannot be reversed and treatment is lifelong.^5^ Thus, the development of regenerative strategies aimed at rebuilding the glaucomatous TM through cell-based approaches could offer substantial benefits to POAG patients.

It has been known for some time that the number of cells residing within the TM decreases with age but also in connection with POAG and other types of glaucoma.^6–8^ The physiologic effects of decreased TM cellularity have been difficult to examine, but there is substantial evidence that dysfunction and degeneration of TM cells is an important factor contributing to elevated IOP in POAG patients (see prior review^9^). These experimental difficulties are due, in part, to the fact that many rodent models of the disease depend upon experimental destruction of the TM and/or episcleral outflow pathways. Such models have proven to be enormously beneficial in the study of retinal or optic nerve outcomes of elevated IOP, but do not allow investigations into the pathophysiology of the anterior eye in glaucoma.^10^ We previously developed a mouse model of glaucoma that constitutively expresses a transgene containing human myocilin with the pathogenic Y437H mutation (Tg-MYOC^Y437H^).^11^ Beginning at the age of 4 months, Tg-MYOC^Y437H^ mice develop elevated IOP in concert with a marked reduction in TM cell density.^11–13^ As typically observed in POAG patients, the overall structure of the TM is not grossly altered in these mice, making them a good experimental model for strategies aimed at regeneration of TM function.

We recently presented data to demonstrate that maintaining induced pluripotent stem cells (iPSC) in culture media preconditioned by primary TM cells induces their differentiation into a cell type that resembles TM cells in morphologic and functional aspects.^14^ These cells are referred to herein as iPSC-TM. One advantage of iPSC is that these cells can be derived from readily available cell types of the intended recipient, such as dermal fibroblasts, thereby enabling autologous transplantation and avoiding immune rejection in the recipient.^15^ We further demonstrated that transplantation of these cells into eyes of Tg-MYOC^Y437H^ mice promotes aqueous humor outflow, lowers IOP, and prevents retinal ganglion cell loss.^12^ The improvement of aqueous humor outflow dynamics in iPSC-TM recipients is accompanied by a significant increase in TM cell density in vivo and by pronounced changes in the gene expression profile that are indicative of cell division. These data suggested that functional renewal of aqueous humor outflow regeneration after glaucomatous damage is possible. However, these first experiments were performed using Tg-MYOC^Y437H^ mice that were at an early stage of the disease, displaying only slight disruption of aqueous humor dynamics and a mild decrease in TM cellularity at the onset of the study.

Thus, the question arose whether a rescue effect also can be demonstrated in older mice with more established pathophysiologic changes, including clearly reduced TM cellularity. The answer to this question has important translational implications, since the majority of POAG patients are older and typically already display evidence of TM dysfunction. In this study, we transplanted iPSC-TM cells into the anterior chamber of naive 6-month-old Tg-MYOC^Y437H^ mice with established TM cell loss and IOP elevation. We then monitored the IOP and the aqueous humor outflow facility for the subsequent 12-week period and determined TM cellularity. Finally, transmission electron microscopy (TEM) and Western blot analysis were used to evaluate endoplasmic reticulum (ER) stress in the TM of transplanted eyes. Our data confirmed our earlier findings in this independent cohort of animals, and indicated that cellular therapy through the use of iPSC-TM also may be possible in older individuals and in eyes with established pathophysiology.

## Materials and Methods

### Mice

All experiments were performed in the F1 offspring of a cross between normal SJL/J and transgenic C57BL/6 mice expressing human myocilin with the Tyr437His mutation (Tg-MYOC^Y437H^).^11^ Age-matched wild type (WT) littermates were selected by genotyping and served as controls. Mice did not undergo any manipulation until they reached 6 months of age, and male and female animals were included. Animal studies were done according to the ARVO Animal Statement for the Use of Animals in Ophthalmic and Vision Research, and approved by the University of Iowa Committee for Animal Care and Use.

### Preparation of iPSC-TM

Mouse iPSC-TM cells were produced from B6.Cg-Tg(ACTB-DsRed*MST)1Nagy/J transgenic mice (Jackson Laboratory, Bar Harbor, ME, USA). These animals express dsRed under the control of a universal promoter, providing a mechanism for identification of the transplanted cells. iPSC from these mice were differentiated into iPSC-TM using a protocol previously established by this laboratory.^12,14^ Briefly, mouse iPSC was maintained in Biopsy Media containing MEM-alpha (Gibco, Waltham, MA, USA), 10% inactive FBS (Gibco), and 0.2% primocin (InvivoGen, San Diego, CA, USA) that was conditioned previously by human primary TM cells. The conditioned media then were sterile filtered and iPSC given for 14 days to drive differentiation. iPSC retaining pluripotency were removed using SSEA-1 conjugated magnetic microbeads and MACS LD columns (Miltenyi Biotec, San Diego, CA, USA). Successful purification and quantitation was verified by labeling iPSC-TM cells with PE-SSEA1 antibody (Becton Dickenson, Franklin Lakes, NJ, USA) followed by flow cytometry (LSR II; Becton Dickenson). Only preparations that were completely free of SSEA-1–positive cells were used for injection.

### Anterior Chamber Injection

Mice were sedated using xylazine and ketamine (12.5 mg/ml and 87.5 mg/kg, respectively). iPSC-TM cells were prepared in 3 μl phosphate buffered saline (PBS) and 5 × 10^4^ cells were delivered into the anterior chamber using 10 μl Hamilton syringes (Hamilton, Reno, NV, USA) with 30-gauge ½-inch length sterile needles (Becton Dickenson). Sham control mice received an injection of an equal amount of PBS. Lubricant was applied to avoid excessive dry eyes during anesthesia (GenTeal Severe Dry Eye Relief; Novartis, Basel, Switzerland).

### IOP Measurement

Mice were anesthetized with 2.5% isoflurane. Exactly 5 minutes after exposure to isoflurane, a rebound tonometer was used to measure the IOP (TonoLab; Colonial Medical Supply, Windham, NH, USA), as described previously.^12,16^ All measurements were taken between 9 AM and 12 PM by investigators blinded to the animals' status.

### Aqueous Humor Outflow Facility Measurement

The aqueous humor outflow facility was determined as described previously.^12^ Mice were anesthetized with ketamine and xylazine as described above. A 33-gauge needle (Becton Dickenson) was used to cannulate the anterior chamber. Saline 0.9% was pumped into the eye using a 150 μl Hamilton syringe (Hamilton Company, Reno, NV, USA) mounted on a computer-controlled syringe pump. The pressure in the system was determined using a flow-through pressure transducer (IcuMedical, San Clemente, CA, USA). Measurements from the transducer were monitored by a computer and recorded by HemoLab software (Stauss Scientific, Iowa City, IA, USA). Flow rates required to maintain IOP were recalculated every second and logged. Flow rates determined to sustain 15, 25, and 35 mm Hg were recorded for 10 to 15 minutes each and the outflow facility (μl/min/mm Hg) was calculated by linear regression.

### Transmission Electron Microscopy

Half of each anterior segment was collected, fixed in half-strength Karnovsky's fixative (2.5% glutaraldehyde, 2% formaldehyde), washed with 0.1 M cacodylate buffer, postfixed with 1% aqueous OsO4/0.2 M in cacodylate buffer, and rinsed with cacodylate buffer. After dehydration, tissues were embedded in Epon and sectioned to 70 nm ultra-thin sections. Stained sections on grids were imaged by a transmission electron microscope (JEOL, Peabody, MA, USA). This portion of the study was completed at the Central Microscopy Research Facility at the University of Iowa.

### TM Cellularity Analysis

Anterior segments were dissected carefully under the microscope. Eyes were submerged in 4% paraformaldehyde in PBS for 2 hours, embedded in OCT, and sectioned at 10 μm thickness using a CryoJane Tape-Transfer System (Leica, Buffalo Grove, IL, USA). Nine serial sections from each sample were incubated at 4°C using an antibody directed against dsRed (Abcam, Cambridge, MA, USA) followed by incubation with an Alexa Fluor 546 labeled Goat anti-Rabbit IgG secondary antibody (Thermo Fisher Scientific, Waltham, MA, USA) at room temperature for 2 hours. Nuclei were stained with 0.1 μg/ml 4′,6-diamidino-2-phenylendole (DAPI; Life Technologies, Carlsbad, CA, USA). Photomicrographs were taken on a fluorescence microscope (BX52; Olympus, Tokyo, Japan). On each image two lines were drawn vertically at the anterior and posterior margins of Schlemm's canal using ImageJ (http://imagej.nih.gov/ij/; provided in the public domain by the National Institutes of Health, Bethesda, MD, USA). Nuclei of (dsRed-positive) iPSC-TM cells and total TM nuclei in this frame were counted and averaged. The difference between the number of total TM cell nuclei and iPSC-TM nuclei is assumed to be the number of endogenous TM cells.

### Immunohistochemical Analysis

Sagittal sections from mouse eyes obtained 12 weeks after iPSC-TM injections (or appropriate controls) were prepared as described above. Sections were incubated overnight at 4°C with rabbit antibodies directed against Iba1 (diluted 1:1000; Wako Pure Chemical, Osaka, Japan) as well as hamster anti-TCR α and β chains (diluted 1:250; Abcam). Binding patterns were visualized using species-specific secondary antibodies (Donkey anti-Rabbit IgG [H+L]; Alexa Fluor 546; Life Technologies; Thermo Fisher Scientific) and Goat anti-Hamster IgG H&L Alexa Fluor 488 (Abcam) diluted 1:500 in PBS. DAPI was used to counterstain to sections. A minimum of three sections from each eyes were evaluated.

Ki-67–positive cells were identified using a rabbit anti-mouse primary antibody (EMD Millipore, Billerica, MA, USA) following a 5-minute pretreatment with 1% sodium dodecyl sulfate (SDS). Sections were stained with DAPI to label nuclei and facilitate orientation.

### Western Blot Analysis

The tissue of the iridocorneal angle was isolated under a dissection microscope and lysed in TAP-lysis buffer. After mixing with 4× LDS-PAGE buffer containing 10× reducing agent, 15-20 μg of total protein were separated on SDS polyacrylamide gels by electrophoresis followed by transfer to a nitrocellulose membrane. Dried blots were blocked in Odyssey blocking buffer (LI-COR biosciences, Lincoln, NE, USA) and incubated at 4°C with specific primary antibodies (goat anti-myocilin [sc-21243] 1:500; Santa Cruz Biotechnology, Santa Cruz, CA, USA; Abcam rabbit anti-calnexin [ab133615] 1:1000; Abcam; and anti-GAPDH [DyLight 680 conjugated, GA1R] 1:5000; Thermo Fisher Scientific). The blots were rinsed with PBS/Tween buffer (PBST) and incubated with corresponding IRdye secondary antibodies (LI-COR Biosciences, Lincoln, NE, USA). The proteins then were visualized using the Odyssey CLx system (LI-COR Biosciences). Quantitation was done using ImageStudio software (LI-COR Biosciences).

### Statistical Evaluation

In this study Student's *t*-test was used to evaluate statistical significance between experimental groups. The Pearson correlation test was used to evaluate the relationship between TM cellularity and IOP. Throughout this study a *P* value <0.05 was considered to be significant.

## Results

### Effects of iPSC-TM on Aqueous Humor Outflow

**Figure 1:**
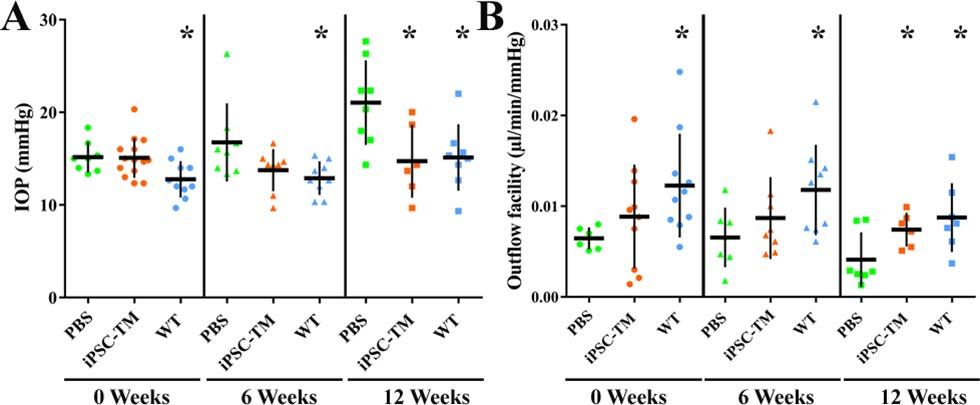
Effects of iPSC-TM on aqueous humor outflow in aged Tg-MYOC mice. IOP (A) and outflow facility (B) were determined in PBS recipient controls, iPSC-TM recipients, and WT mice. Measurements were taken before transplantation (0 weeks), and 6 and 12 weeks after transplantation. *P < 0.05.

As previously published, TM degeneration in Tg-MYOC^Y437H^ mice first becomes apparent at approximately 4 month of age and IOP continues to rise subsequently if no treatment is applied. In this study, aimed to investigate the utility of iPSC-TM transplantation in mice with established TM degeneration, 6-month old Tg-MYOC^Y437H^ mice were used. Transgenic mice then were assigned to a treatment or control group and care was taken to ensure that no significant difference in IOP or outflow facility existed between the groups (Fig. 1A). Both groups of transgenic mice displayed significantly higher IOP when compared to age-matched WT animals. The average pretransplantation IOP of the iPSC-TM recipient group (*n* = 8) was 15.17 ± 1.66 mm Hg, whereas the sham injection group (*n* = 14) had an average IOP of 15.08 ± 2.15 mm Hg. The naïve control group (*n* = 10) had an IOP of 12.77 ± 2.00 mm Hg (Fig. 1A). Correspondingly, aqueous humor outflow facility, a measure of TM-mediated conventional outflow, is decreased in the Tg-MYOC^Y437H^ mice used for PBS or iPSC-TM treatment when compared to WT mice (0.00645 ± 0.0012 and 0.00885 ± 0.0057 vs. 0.012 ± 0.0057 μl/min/mm Hg; Fig. 1B). These data confirm that 6-month-old Tg-MYOC^Y437H^ mice have established aqueous humor outflow disturbances and are a viable mouse model to explore the effect of iPSC-TM on TM restoration.

The treatment group received 50,000 iPSC-TM cells by a single injection into the anterior chamber while additional Tg-MYOC^Y437H^ mice were sham injected with PBS. A group of age-matched WT littermates were used as naïve controls. IOP and outflow facility were measured 6 and 12 weeks after transplantation. At 6 weeks after transplantation, iPSC-TM recipients exhibited a reduction of IOP and slight improvement of outflow facility when compared to values in PBS recipients, but these data did not reach statistical significance (13.75 ± 2.27 mm Hg vs. 16.75 ± 4.22 mm Hg; 0.0087 ± 0.0045 μl/min/mm Hg vs. 0.0066 ± 0.0033 μl/min/mm Hg). In the following weeks, IOP and outflow facility continued to degenerate in sham-injected eyes, whereas iPSC-TM recipients continued to recover. At 12 weeks after transplantation, the IOP in iPSC-TM receipts resembled that of WT mice (14.72 ± 3.94 vs. 15.13 ± 3.59 mm Hg) and was significantly lower than that observed in PBS receipts (21.02 ± 4.57 mm Hg; *P* = 0.019). Likewise, aqueous humor outflow facility in iPSC-TM receipts was markedly higher than that of PBS receipts (0.0074 ± 0.0019 vs. 0.0041 ± 0.003 μl/min/mm Hg; *P* = 0.004). At this point the outflow facility of iPSC-TM recipients was similar to that of WT mice (0.0087 ± 0.0038 μl/min/mm Hg). These findings indicated that transplantation iPSC-TM is not only an effective approach to maintain normal aqueous humor outflow in older Tg-MYOC^Y437H^ mice, but also to reverse existing deficits.

### TM Immunohistochemistry

**Figure 2:**
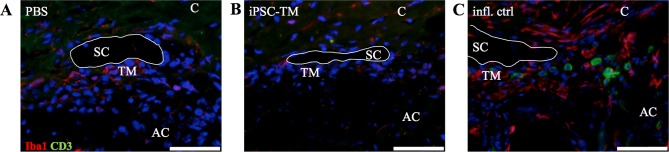
Immunohistochemical detection of Iba1 or CD3 in sagittal sections of Tg-MYOC^Y437H^ mice having received either injections of (A) PBS, or (B) iPSC-TM. Both antigens are readily detectable in eyes from a mouse that developed ocular inflammation following an injury (C). Sections are counterstained with the nuclear dye DAPI and Schlemm's canal is outlined to facilitate orientation. C, cornea; AC, anterior chamber; SC, Schlemm's canal. Scale bars: 50 μm.

The development of an immune response to the transplanted material could potentially result in the release of signaling molecules that increase aqueous humor outflow through the uveoscleral pathway and lower IOP. Throughout the study, visible signs of inflammation were not observed in any of the iPSC-TM or PBS-injected eyes. In addition, we performed an immunohistochemical study to determine if CD3-positive cells or Iba1-positive macrophages or microglia infiltrate the TM in transgenic mice (*n* = 4 eyes/group). Our findings demonstrated that, while Iba1+ cells can be detected at a low frequency in all examined sections, they do not appear to be more numerous in iPSC-TM recipients than in control mice (Figs. 2A, 2B). CD3+ cells are essentially absent from the trabecular meshwork in all examined sections. In contrast, Iba1 and CD3 immunoreactivity is markedly higher and readily apparent in a mouse eye, used here as a positive control, with inflammation resulting from an injury (Fig. 2C). These findings indicated that infiltration of immune cells is not induced by transplantation of iPSC-TM derived from mice with the same genetic background as the recipients.

### Cellular Effects of iPSC-TM Cells

**Figure 3:**
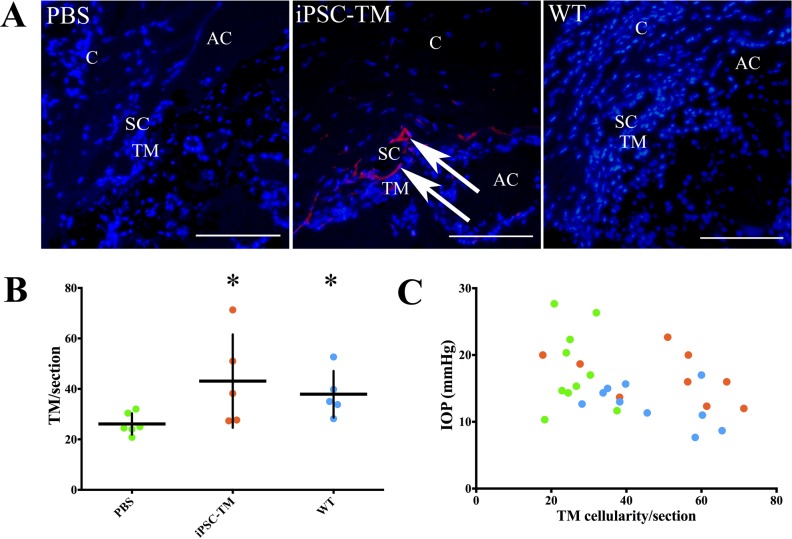
Effects of iPSC-TM transplantation on TM cellularity. (A) Immunohistochemical evaluation of anterior segments of PBS and iPSC-TM recipients, and WT mice to detect iPSC-TM expressing dsRed (red, arrows). DAPI (blue) was used to label nuclei. Scale bar: 100 μm. (B) Endogenous TM cells in PBS and iPSC-TM recipients, and WT mice were exhibited. *P < 0.05. (C) Correlation analysis of IOP and total TM cellularity using all samples from PBS and iPSC-TM recipients, and WT mice.

At 12 weeks after transplantation, dsRed-positive iPSC-TM cells were occasionally visible on sagittal sections of the iridocorneal angle of transplanted eyes. To determine how frequently these cells survive and whether an effect on endogenous TM cells can be detected, we performed a morphometric study using eyes from PBS recipients (*n* = 6), iPSC-TM recipients (*n* = 5), and WT mice (*n* = 5). From each eye, nine sections were prepared and dsRed-positive iPSC-TM cells and the eyes' native dsRed-negative TM cells were counted (Fig. 3A). In accordance with previous reports, the number of endogenous TM cells was significantly lower in PBS receipts when compared to age-matched WT controls (26.1 vs. 37.8 cells/section; *P* = 0.01) (Fig. 3B). Transplantation of iPSC-TM cells increased overall TM cellularity in most Tg-MYOC^Y437H^ mice considerably (43.1 cells/section; *P* = 0.028) and resulted in cellular density similar to that of WT mice. However, in these iPSC-TM recipients, only 0.9 dsRed-positive iPSC-TM cells were detected on each section (<1/section). While these data demonstrated that some iPSC-TM survive in the anterior segment at least after 3 months after transplantation, they also suggest that the increased cellularity in iPSC-TM recipients results from additional endogenous cells rather than large numbers of implanted iPSC-TM.

To further understand the relationship between TM cellularity and IOP, a scatter plot was generated by charting these values from each individual eye included in the study. As shown in Figure 3C, the number of TM cells observed per section ranged from 18.3 to 37.5 in PBS recipients, and the IOP varied from 10.3 to 26.3 mm Hg. WT mice contained 28.2 to 65.5 TM cells, and their IOP ranged from 7.67 to 17 mm Hg. Finally, in iPSC-TM recipients, the number of TM cells ranged from 17.7 to 75.8, and IOP varied between 12.0 and 22.67 mm Hg.

The correlation between TM cellularity and IOP in all datasets was examined using Pearson's correlation test. The result demonstrated a strong correlation between these two parameters (*P* = 0.044) and suggested that it is feasible to regulate IOP through increasing TM cellularity, at least in Tg-MYOC^Y437H^ mice. However, these findings did not resolve whether the observed correlation is due to a direct activity of iPSC-TM, for example, contractility, or indirect effects, for example, alteration in the biophysical properties of the TM.

**Figure 4:**
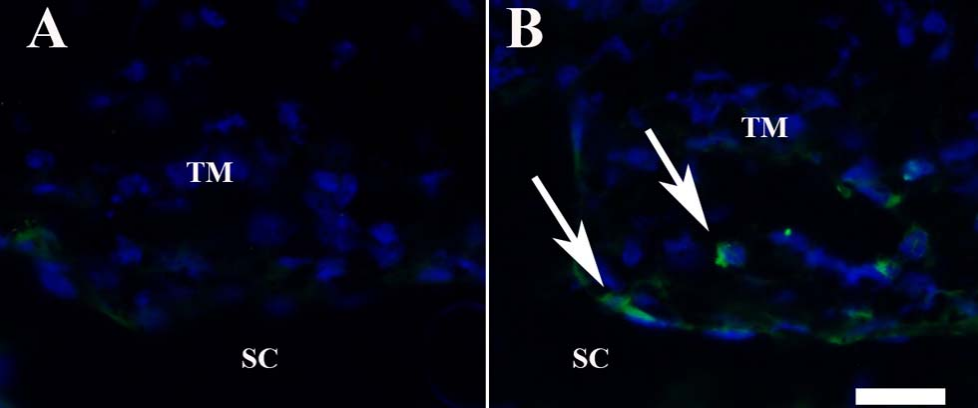
Immunohistochemical detection of Ki-67 antigen in the trabecular meshwork of (A) PBS injected and (B) iPSC-TM recipient Tg-Myoc mice. Ki-67–positive cells (green, arrows) can be readily detected in iPSC-TM recipients, but are rare in the TM of control eyes. Nuclei are counterstained with DAPI (blue). Scale bar: 25 μm.

Our assumption that the higher number of TM cells observed in iPSC-TM recipient eyes is the result of cell division of the recipients' endogenous TM cells is based on our previous data demonstrating elevated expression of numerous genes associated with cell division in the TM of MYOC^Y437H^ mice after iPSC-TM transplantation in vivo. Furthermore, we observed that coculture with iPSC-TM enhances the growth rate of primary TM cells in vitro. To provide direct evidence of cell division in the TM after iPSC-TM transplantation in vivo, we performed an immunohistochemical analysis of Ki-67–positive cells in eyes of iPSC-TM recipients, PBS recipients, and WT control mice (*n* = 4/group). This antigen is a well-established morphologic marker of dividing cells and is absent in resting (G_0_) cells.^17,18^ In our analysis, Ki-67–positive cells were rare in the TM of WT or PBS-injected mice, although the antigen could be detected in other portions of the eye, such as the corneal epithelium. In contrast, Ki-67–positive cells are observed frequently in the TM of iPSC-TM recipient eyes, indicating that these cells are mitotically active (Fig. 4). None of the detected cells exhibiting nuclear or perinuclear Ki-67 labeling was found to express dsRed, indicating that these cells are not transplanted iPSC-TM.

### Effects of iPSC-TM Transplantation on ER Structure and ER Stress

**Figure 5:**
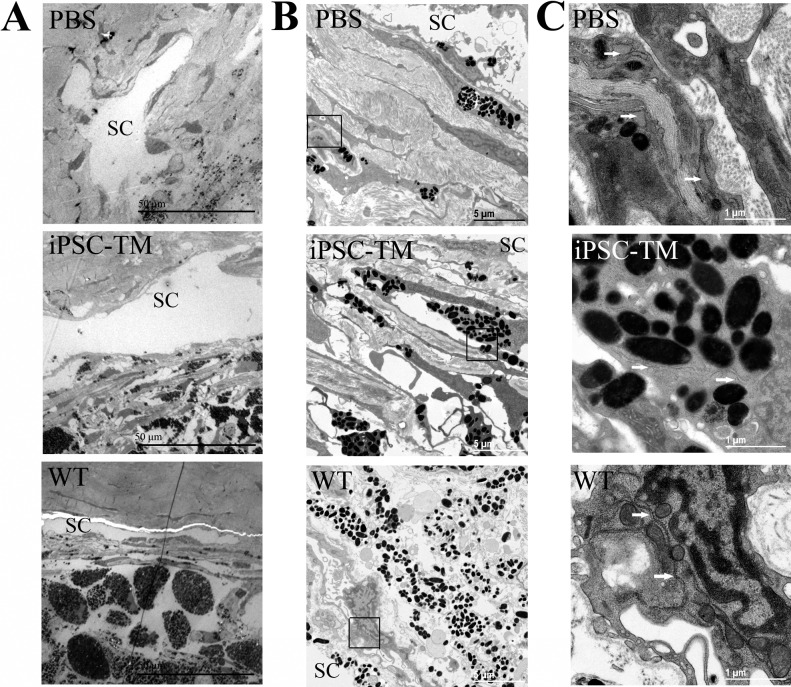
Ultrastructural appearance of TM in aged Tg-Myoc mice having received sham (PBS) injections (top row), iPSC-TM injections (middle), or in age-matched WT mice (bottom) as observed by TEM. Overview of TM tissues (A), individual TM cell (B), and organelles within TM cells (C) were observed in PBS and iPSC-TM recipients, and WT mice. Scale bars: 50, 5, and 1 μm, respectively. Yellow boxes indicate the areas depicted at higher magnification. Arrows indicate ER.

To explore effects of iPSC-TM transplantation on the ultrastructure of TM cells in vivo, eyes were harvested from PBS and iPSC-TM recipients, and WT mice (*n* = 2 each). and evaluated by TEM. Samples were obtained 12 weeks after transplantation. When observed at lower magnification (Fig. 5A) the structures of the iridocorneal angle, including cornea, ciliary body, Schlemm's canal, and TM, appeared largely undisturbed in all animals, including PBS recipients, iPSC-TM recipients, and WT mice. The TM is populated with cells and displays connective beams and lamellae. However, in PBS-treated eyes, fusion of adjacent trabecular beams, shrinking of the intertrabecular spaces, and some loss of overall structure is frequently apparent. Furthermore, when imaged at higher magnification, allowing observation of individual cells and their organelles, TM cells of PBS-injected eyes often displayed degraded nuclei, distended ER, and abnormal mitochondrial morphology with fewer and disorganized membranes (Fig. 5B). In contrast, TM cells in iPSC-TM recipient mice displayed regular nuclei, normal rough ER, and organized mitochondria very similar to those observed in WT eyes. Thus, transplantation of iPSC-TM cells maintains or restores TM structure and TM cell organelles in older Tg-MYOC^Y437H^ mice.

**Figure 6:**
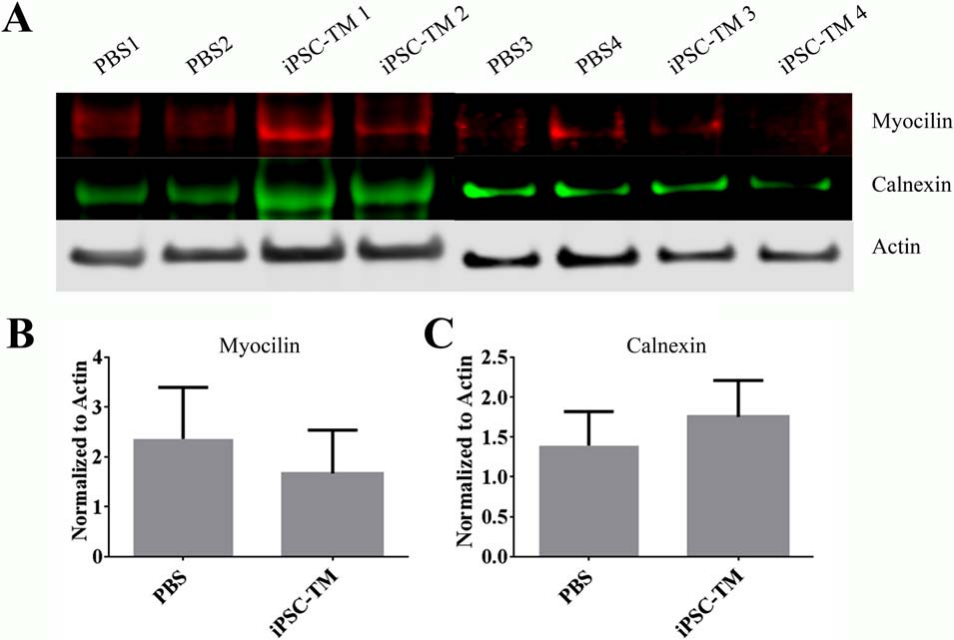
Western blot analysis of ER stress-related proteins in the iridocorneal angle of aged Tg-Myoc mice. (A) Myocilin and calnexin expression in PBS recipients and iPSC-TM recipients. Beta-actin was used as a loading control (B). Expression levels of myocilin normalized to β-actin demonstrating continued expression of the transgene. Normalized values are 2.36 and 1.67 in PBS and iPSC-TM recipients, respectively (P = 0.34). (C) Expression levels of calnexin normalized to β-actin. Expression ratios are 1.39 and 1.75 in PBS and iPSC-TM recipients, respectively (P = 0.29).

To further investigate the biological effects of iPSC-TM transplantation on ER stress, we determined the levels of myocilin and calnexin in TM tissues obtained from PBS (*n* = 4) and iPSC-TM (*n* = 4) recipients 12 weeks after injection by Western blot analysis. Misfolding of myocilin and retention in the ER leads to a profound Unfolded Protein stress response in TM cells and is a cellular hallmark associated with TM dysfunction in this model. Calnexin was used as a cellular marker for ER stress-induced apoptosis.^19^ Here, myocilin and calnexin are readily detectable in TM homogenates of Tg-MYOC^Y437H^ mice, whether or not they received iPSC-TM or PBS injections (Fig. 6). To compare these levels more precisely, the expression levels from each sample were quantified and normalized to β-actin expression. The resultant data demonstrated that the normalized expression levels of myocilin in PBS and iPSC-TM recipients were 2.36 and 1.67, respectively (*P* = 0.34). Similarly, the average levels of calnexin in these two groups were 1.39 and 1.75 (*P* = 0.29). Consequently, it appears unlikely that iPSC-TM transplantation into Tg-MYOC^Y437H^ mice reduces myocilin intracellular retention or has a lasting effect on ER stress in the TM.

## Discussion

The health of the TM is crucial for the regulation of IOP.^20^ We previously demonstrated that, in the Tg-MYOC^Y437H^ mouse model of glaucoma, transplantation of iPSC-TM reverses the decline of aqueous humor outflow facility and lowers IOP.^12^ However, the mice used in our first study were relatively young and represented an early stage of disease. It remained unclear whether transplantation also is effective in older animals that display more damage to TM structure and cells. This is a particularly relevant consideration, since a large fraction of POAG patients present with moderate or severe pathology.^21,22^ To address this question, we transplanted iPSC-TM into an independent cohort of 6-month-old Tg-MYOC^Y437H^ mice with established aqueous humor outflow deficiencies and elevated IOP.

The data obtained in this study demonstrated that, as in younger animals, iPSC-TM transplantation into older mice does result in heightened aqueous humor outflow facility, lower IOP, and increased TM cellularity. These outcomes demonstrated that TM regeneration also can be achieved in eyes with clearly established pathology. At the same time, subtle differences in the responses of these older mice were apparent. In younger mice aqueous humor outflow facility was restored to normal levels within 6 weeks, whereas older mice did not display statistically significant results in the same period. In the group of older mice, significant improvement required additional time but could be demonstrated 12 weeks after transplantation. It also is noteworthy that older mice displayed a higher variance in their response than observed in younger animals. Finally, a statistically significant preservation of retinal ganglion cells could not be demonstrated in this cohort (data not shown). The reasons for this remain unclear, but it is conceivable that IOP independent factors contributing to glaucoma damage become more prominent in older mice. It is equally possible that the comparatively short period of reduced IOP (less than 6 weeks in 9-month-old mice) was insufficient to reveal a reduction in the rate of ganglion cell degeneration.

The reason for the slightly reduced IOP and aqueous humor outflow response to iPSC-TM transplantation in older mice also remains speculative. We previously argued that the increase in TM cellularity is due to proliferation of endogenous TM cells rather than implantation of iPSC-TM.^11,12^ This notion is supported further by our current findings demonstrating few implanted iPSC-TM within the TM, but evidence for TM cell division. Since the number of surviving endogenous TM cells is lower in 6-month-old than in 4-month-old Tg-MYOC^Y437H^ mice, one explanation for the delayed reestablishment of normal outflow may be that additional cell division is required to achieve normal TM cellularity. The delay may be further compounded by a reduced ability of older cells to proliferate,^23^ although it must be noted that the 6-month-old mice used here are not nearing senescence.^24^ It is equally likely that reduced outflow facility in Tg-MYOC mice is due to accumulating structural or physical changes to the TM that are secondary to diminished TM cell function. Progressive changes, such as stiffening of the TM or a reduction in autophagy, are associated with POAG and may be related to reduced matrix turnover and phagocytosis by TM cells.^25–29^ While such changes to the TM have not yet been investigated in this disease model, it is likely that TM pathology becomes more difficult, and slower to reverse as the disease becomes established in older individuals.

One concern of transplantation studies is that it is conceivable that the observed reduction of IOP is the result of an immune reaction in the TM brought about by transplantation itself. Inflammation temporarily increases uveoscleral outflow (see prior review^30^) and, thus, has the potential to lower IOP and obfuscate the interpretation of results. In our study, inflammation in the anterior chamber was unlikely to develop since iPSC-TM were derived from mice with the same strain and genetic background, including MHC self-antigens, as the recipients. The notion is supported further by our immunohistochemical data indicating that transplanted eyes, harvested 12 weeks after transplantation when improved aqueous humor dynamics can be observed clearly, do not exhibit increased infiltration of leukocytes of macrophages. It also is important to note that humor outflow facility is a measure of TM-mediated conventional outflow, rather than uveoscleral flow.^31^ Finally, increased TM function following iPSC transplantation also has been reported by others in an in vitro system that precludes the development of an inflammation response.^32^

While most eyes that received iPSC-TM displayed a robust increase in TM cellularity, fewer than 1% of the cells within the TM were identified as iPSC-TM. These findings are similar to our previously published data demonstrating that few iPSC-TM integrate or survive into the TM of Tg-MYOC^Y437H^ mice with early glaucomatous changes.^12^ Based on in vitro studies and in vivo gene expression data, we had suspected previously that iPSC-TM induce cell division of endogenous TM cells in these young eyes. This hypothesis is supported further by our current findings of Ki-67–positive cells in the TM of iPSC-TM recipients. These data provided evidence of cell division in vivo and demonstrated that ability of TM cells to divide is not lost in the older animals used here. It also is interesting to note that the proliferative burst of the endogenous TM cells does not lead to excessive TM cellularity. In this and our earlier study, TM cellularity in transplanted eyes was similar to that of age-matched WT control mice. This suggested that feedback signals exist between TM cells, or perhaps the niche and the cells, that prevent the development of outflow deficits due to cellular overcrowding.^33,34^

We had suspected previously that one reason for the low frequency of implanted iPSC-TM may be that, in younger mice, TM cellularity still is reasonably high and that a niche for implantation of iPSC-TM may not be available. Similar observations have been made in graft survival studies in other disease models.^33,35^ Lack of niche is less likely to affect implantation in the older mice, since they experienced a clear reduction in TM cell number at the beginning of the experiment.

Alternatively, it is conceivable that injected iPSC-TM do not implant into the TM, either due to cell adhesion inhibition from endogenous cells or that the iPSC-TM adjusts poorly to the microenvironment in vivo. Both of these conditions could result in poor graft survival^33^ but could potentially be overcome by a number of preconditioning approaches, including pretreatment with growth factors,^36^ modifying cell viability through overexpression of anti-death signals,^37,38^ or hypoxic preconditioning of implanted cells.^39^ While largely untested in iPSC, similar strategies may be beneficial to enhance graft survival of iPSC-TM to achieve robust and reproducible improvements in aqueous humor dynamics in glaucomatous eyes.

One of the hallmarks of the pathogenic MYOC^Y437H^ myocilin mutation is the accumulation of misfolded protein in the ER, leading to ER stress in TM cells, and eventually cell death.^11,40–42^ Morphologic indications of this process are commonly evident by TEM in 9-month-old Tg-MYOC^Y437H^ mice and include distended ER, disorganized mitochondria, and degraded nuclei in TM cells. In contrast, the organelle structure in the TM of Tg-MYOC^Y437H^ mice after iPSC-TM was improved dramatically and very similar to that observed in age-matched controls. Given the fact that even in transplanted animals the vast majority of resident TM cells are not the iPSC-TM derived from cells of normal mice, but endogenous TM cells expressing MYOC^Y437H^, these findings are perhaps surprising. It is reasonable to assume that cell division would reduce the amount of aggregated myocilin in each cell, thereby reducing the level of ER stress, but it also is possible that other mechanisms are evoked that lead to a reduction of cell stress. Our subsequent Western blot analyses indicated that myocilin is expressed at similar levels in iPSC-TM recipients and transgenic control animals, and that calnexin, a marker of ER stress,^43^ accumulates at similar levels in both groups. These data indicated that ER stress continues to affect TM cells in this model, even after iPSC-TM transplantation and replication of endogenous TM cells, as can be expected due to the constitutive expression of transgenic MYOC^Y347H^ under the control of the CMV promoter.^11^

One explanation to reconcile the seemingly contradictory findings of improved TM ultrastructure despite continued MYOC^Y347H^ expression is that some degree of myocilin misfolding and ER stress can be tolerated in TM cells and does not lead to overt morphologic indications, at least within the study period. If this is the case, one might expect the newly replicated TM cells to eventually display signs of pathology leading, perhaps, to cell death and the reemergence of TM dysfunction. This would further suggest that TM cellularity is a more important factor in TM dysfunction than ER stress status. The importance of TM cellularity is highlighted further by the correlation between IOP and TM cell density. However, it must be pointed out that these data did not implicate a mechanism or prove that the activity of TM cells is directly affecting outflow facility, for example, through enhanced cell-mediated TM contraction.

To which degree our findings might translate to human disease is difficult to predict. Our animal model relied on expression of MYOC^Y347H^ which, in humans, causes elevated IOP and glaucoma as early as in the second decade of life.^44^ Since the observed rescue effect appears to rely on replication of endogenous TM cells, along with any genetic variations these might harbor, it is possible that iPSC-TM transplantation has a transient effect in eyes that have elevated IOP due to a strong disease-causing mutation. However, the effects of iPSC-TM transplantation were maintained for at least 3 months in Tg-MYOC^Y437H^ mice, which correlates to approximately 10 years in humans.^24,45^ Additionally, the vast majority of POAG patients are not afflicted with strong genetic factors and their TM remains functional for many decades before aqueous humor outflow deficiencies develop. As such, we suspected that in these individuals the newly derived cells will survive for an extended period of time and that restoration of function will have a more sustained effect.

In conclusion, our data obtained in an independent cohort of animals confirmed our previous findings that transplantation of iPSC-TM can restore aqueous humor outflow and demonstrated that this also is possible in eyes with established glaucoma pathology. The restorative effect is very likely due to the proliferation of endogenous TM cells following stimulation by transplanted iPSC-TM. These findings are encouraging and suggest that stem cell–based regeneration of the TM in POAG may be possible.
